# Is musical engagement enough to keep the brain young?

**DOI:** 10.1007/s00429-022-02602-x

**Published:** 2022-12-27

**Authors:** Anna Maria Matziorinis, Christian Gaser, Stefan Koelsch

**Affiliations:** 1 Department of Biological and Medical Psychology, University of Bergen, Jonas Lies Vei 91, 5009 Bergen, Norway; 2 Department of Neurology, Jena University Hospital, Am Klinikum 1, 07747 Jena, Germany

**Keywords:** Machine-learning, Brain aging, Brain Age Gap Estimation, Musical training, Active musical engagement, Resilience

## Abstract

Music-making and engagement in music-related activities have shown procognitive benefits for healthy and pathological populations, suggesting reductions in brain aging. A previous brain aging study, using Brain Age Gap Estimation (BrainAGE), showed that professional and amateur-musicians had younger appearing brains than non-musicians. Our study sought to replicate those findings and analyze if musical training or active musical engagement was necessary to produce an age-decelerating effect in a cohort of healthy individuals. We scanned 125 healthy controls and investigated if musician status, and if musical behaviors, namely active engagement (AE) and musical training (MT) [as measured using the Goldsmiths Musical Sophistication Index (Gold-MSI)], had effects on brain aging. Our findings suggest that musician status is not related to BrainAGE score, although involvement in current physical activity is. Although neither MT nor AE subscales of the Gold-MSI are predictive for BrainAGE scores, dispositional resilience, namely the ability to deal with challenge, is related to both musical behaviors and sensitivity to musical pleasure. While the study failed to replicate the findings in a previous brain aging study, musical training and active musical engagement are related to the resilience factor of challenge. This finding may reveal how such musical behaviors can potentially strengthen the brain’s resilience to age, which may tap into a type of neurocognitive reserve.

**Supplementary Information:**

The online version contains supplementary material available at 10.1007/s00429-022-02602-x.

## Introduction

The global aging population is increasing along with a concurrent rise in the incidence of neurodegenerative diseases. The additional social burden and economic implications of this rise have revealed the need to explore the efficacy of potential brain-sparing activities that can aid in the maintenance of cognitive health. Healthy aging is typically accompanied by decreasing cognitive functioning, including declining processing speed, inhibition, attention, working and episodic memory, semantic fluency, visuospatial and visuoconstructional abilities, as well as difficulties with executive functioning, and social understanding (Borella et al. 2008; Salthouse 2010; Hartshorne and Germine 2015; Fjell et al. 2017; Sutcliffe et al. 2020). Healthy brain aging is characterized by a decline in brain volume over time and can be determined via structural magnetic resonance imaging (MRI). A meta-analysis of 52 longitudinal studies revealed brain volume atrophy including decreasing gray and white matter volume (Hedman et al. 2012). Further, an Enhancing Neuroimaging Genetics through Meta-Analysis (ENIGMA) Consortium study assessed subcortical volumes from 18,605 individuals across the lifespan and found that the thalamus, hippocampus, and amygdala begin to decline in the sixth decade of life, and enlargements of the lateral ventricles infused with increasing amounts of cerebral spinal fluid are found over the life course (Dima et al. 2021). Studies quantifying the effects of age on brain volume have used volumetric analysis as a means to predict the risk of developing neurodegenerative disease. Brain Age Gap Estimation, or BrainAGE, is one such model that takes into account healthy brain maturation data from T1-weighted structural MRI scans and models brain age estimates based on relevance vector regression (Franke et al. 2010).

Music-based activities, such as music therapy, music-making or training, or mere active engagement in music have been shown to improve cognition, emotion, and well-being among older adults (Piccirilli et al. 2021), yet how musical training affects brain plasticity and brain efficiency is still under investigation. The aim of the current study is to inquire if a background of music-making/musical training or current active musical engagement can be protective for brain aging in healthy adults as measured by a brain aging algorithm, namely BrainAGE. Additionally, the current study aims to replicate the findings from a previous brain aging study (Rogenmoser et al. 2018) and to test if in the absence of musical training, if active musical engagement alone is sufficient to produce an age-decelerating effect in healthy adults.

### Musical training and music-making

Due to the multi-modal applications of musical training on the brain, musical expertise has been recognized to be a valuable model for studying structural brain plasticity mechanisms (Wan and Schlaug 2010), specifically training-induced neuroplasticity. Musical training is related to structural and functional alterations in networks throughout the brain as music is able to engage various systems such as the auditory, visual, motor, memory, attention, emotion, and reward networks (Sutcliffe et al. 2020). Consequently, active engagement in music can increase demand on brain networks by stimulating multi-sensory integration, learning, reward processing, and cognition as a means to bolster brain activity in adults (Sutcliffe et al. 2020). The rewarding nature of music can promote further musical learning, and further drive neuroplastic changes associated with musical training (Penhune 2019). Furthermore, musical training has protective effects by enhancing cognitive functioning in healthy aging (Abrahan et al. 2019) and building the brain’s resilience to age (Hanna-Pladdy and Gajewski 2012). Effects on structural brain plasticity have been found throughout the lifespan (Hyde et al. 2009; Wan and Schlaug 2010), and the degree of plasticity changes are highly variable and typically reduce with age (Pauwels et al. 2018). It has been suggested that music-related increases in brain volume and structural connectivity can offset age-related cognitive decline and may delay the onset of clinical symptoms related to neurodegeneration (Fauvel et al. 2013). Musicians appear to be less susceptible to age-related deterioration in the brain (Sluming et al. 2002), showing neurocognitive advantages in tests of reasoning (Brandler and Rammsayer 2003) perceptual speed (Helmbold et al. 2005), visual and verbal working memory (Berti et al. 2006; George and Coch 2011; Hanna-Pladdy and Gajewski 2012), as well as a preservation of inhibitory regulation (Amer et al. 2013; Grassi et al. 2017; Moussard et al. 2016). Although high-activity musicians reap additional benefits in visuospatial abilities in relation to low-activity musicians (Strong and Mast 2019), research suggests that regardless of activity status, musicians show preserved cognitive functioning in advanced age (Hanna-Pladdy and MacKay 2011). Overall, musical training seems to be positively associated with neurocognitive benefits.

Moreover, music-making, which is regarded as an active process of playing and making music, is generally accepted as a brain-sparing activity that has shown promise in improving neurocognitive health. A meta-analysis exploring the effects of musical practice in cognitively healthy individuals aged 59 years or older found that music-making had positive effects on processing speed, attention, verbal working memory, and inhibition (Román-Caballero et al. 2018). A review by Särkämö investigated the influence of musical activities (e.g., playing, singing, listening, and dancing) and results showed overall positive effects on brain aging, specifically in dementia and stroke populations (Särkämö 2018). Another review (Sutcliffe et al. 2020) found that although music-making may show potential benefits in neuropsychological aging, various methodological concerns have been cited such as low sample sizes, lack of appropriate control groups, and longitudinal interventions. The claims that music-making may have plasticity effects on the aging brain needs further investigation.

### Brain Age Gap Estimation or BrainAGE

Modeled as a neuroimaging biomarker of age-related deterioration, Brain Age Gap Estimation, or BrainAGE for short, is a well-validated (Franke and Gaser 2012) machine-learning algorithm, and is used as a marker of accelerated brain atrophy (Franke et al. 2010). BrainAGE was developed using a machine-learning pattern recognition method, specifically relevance vector regression (RVR) (Tipping 2001) to provide a brain age metric known as the brain age gap (BAG) reflecting the differences between chronological (biological) and estimated brain ages. The larger the difference between these estimates, the more age-accelerating (positive scores) or age-decelerating (negative scores) the interpretation of the estimate.

The BrainAGE method was the first to establish reference curves for healthy brain maturation from childhood to older age (Franke et al. 2010) and has validated its use with regards to brain maturation in children and adolescents (Franke et al. 2012; Franke and Gaser, 2019)⁠. The BrainAGE method has effectively examined aberrant aging resulting in predictions of worsening cognitive functions and possible conversions to serious neurodegenerative diseases (Löwe et al. 2016; Gaser et al. 2013; Franke and Gaser 2012)⁠. Due to its sensitivity, BrainAGE has outperformed biomarkers based on cerebrospinal fluid (e.g., Amyloid beta (Aβ42) total and phosphorylated tau) and has shown its ability to predict aberrant progression from Mild Cognitive Impairment to Alzheimer’s Disease (AD) (Gaser et al. 2013). A study revealed that the prediction of conversion to AD was more accurate using BAG compared to neuropsychological test scores, even when Apolipoprotein E (APOE) status was unknown (Löwe et al. 2016)⁠.

BrainAGE has been used across various domains including in metabolic disorders such as type 2 diabetes mellitus (Franke et al. 2013) and obesity (Mcwhinney et al. 2021), psychiatric clinical populations such as schizophrenia and bipolar disorder (Nenadić et al. 2017), and has been investigated in relation to certain lifestyle factors like meditation, music-making, smoking, physical activity, alcohol consumption, and social integration (Luders et al. 2016; Rogenmoser et al. 2018; Bittner et al. 2021). In terms of the effects of music-making on BrainAGE, Rogenmoser and colleagues (Rogenmoser et al. 2018) conducted a study in amateur and professional musicians and found larger negative BAG in musicians compared to healthy controls, citing the largest age-decelerating effects in amateur-musicians. Both amateur and professional musicians had an estimated 4 years younger appearing brains than the healthy controls, who showed no difference in terms of their chronological and brain-estimated age.

### Current study aims and hypotheses

The present study explores the role of two different types of musical behaviors on brain aging in healthy adults. We administered questions from the frequently used, and well-validated self-report Goldsmiths Musical Sophistication Index (Gold-MSI v1.0, 11 October 2012), which captures several aspects of musical experience. The Gold-MSI has been shown to be suitable for assessing musicality in non-professional musicians (Baker et al. 2020), and has sufficient sensitivity to be used in professional musician groups and in pathological populations (e.g., amusics) (Müllensiefen et al. 2014). The Gold-MSI reflects inherent or implicit musical skills related to the notion of “musical sleepers” (Law and Zentner 2012) (i.e., individuals listeners with more adept listening skills). Music instructs the perceptual system via exposure, and has shown top-down interactions from cortical to subcortical areas, instructing the perceptual system to better encode the features of the incoming stimulus (Kraus and Chandrasekaran 2010).

For the purpose of our study, we used two subscales from the Gold-MSI, namely musical training (MT) and active engagement (AE), in addition to selected questions from the Emotions subscale, which we used to compute an additional measure of musical pleasure, called Musical Anhedonia (MA). We wanted to measure how musical training and active engagement affect healthy brain aging. We hypothesized that participants with higher levels of musical training would reflect more negative BAG as compared to those with less musical exposure. The same thinking was applied to those with higher levels of active engagement.

Lastly, exploratory analyses were undertaken investigating the relationship between psychological resilience and the aforementioned study measures (BrainAGE, MT, AE, MA). In specific, we used the Dispositional Resilience Scale (DRS-15, Hystad et al. 2010) to assess “hardiness”, which is defined as a combination of personality traits that function as a resilience resource during and after the encountering of potentially traumatizing life events (Kobasa et al. 1983).

## Materials and methods

### Participants

125 healthy subjects (55 males and 70 females, mean age of 53.04 years (SD = 17.13) ranging between the ages of 22 and 89 years) provided signed informed consent. Demographic information can be found in Table 1. Exclusion criteria included (1) a history of psychopathology, (2) traumatic brain injury, (3) neurological illness, (4) sensory disorders (including hearing impairment), (5) vascular disorders, (6) claustrophobia or the (7) presence of ferromagnetic metal in the soft tissue of the body incompatible with MR scanning.

**Table 1:** Demographic characteristics

Demographic characteristics	Non-musicians (*N* = 61)	Amateur-musicians (*N* = 64)
Age	54.36 (18.06)	51.78 (16.24)
Gender (female/male)	35/26	35/29
Handedness (right/left)	58/3	57/7
Years of education	14.61 (2.98)	16.17 (3.43)
Medication (no/yes)		
Blood pressure	54/7	56/8
Anxiety	61/0	63/1
Cholesterol	56/5	61/3
Allergy	57/4	58/6
Metabolism	55/6	61/3
Antidepressants	59/2	60/4
Singing alone (no/yes)	18/43	3/61
Singing in public (no/yes)	38/23	17/47
Dance (no/yes)	21/40	20/44
Currently physically active (no/yes)	14/47	14/50
Grew up with music in the home (no/yes)	18/43	6/58
Parents sung lullabies (no/yes)	16/45	11/53

Listed are the demographic descriptive statistics including means (M), standard deviation (SD), and sample size (n)

### Music questionnaire

We administered the Goldsmiths Musical Sophistication Index (Gold-MSI), v1.0, 11 October 2012 (https://media.gold-msi.org/test_materials/GMS/docs/gms_documentation_en.pdf) for our study. The Gold-MSI is a validated self-report scale that inspects various aspects of musical experience across five subscales (e.g., Active Engagement, Perceptual Abilities, Emotions, Singing Abilities, and Musical Training). The current study selected items using the Gold-MSI configurator (https://shiny.gold-msi.org/gmsiconfigurator/). Selected questions included three questions from the Emotions subscale and two full subscales, namely active engagement and musical training for a total of 19 questions. See Suppl. Mat. for full musical background questionnaire.

The AE subscale is defined as the level of musical engagement including reading, writing, internet activity, listening, and income spent on music and music events attendance. The MT subscale is defined as the level of musical dedication according to time (peak hour per day and amount of training in years) spent on musical training and number of instruments played, including voice (Müllensiefen et al. 2014). Both subscales are closely associated with motor and somatosensory activities (Müllensiefen et al. 2014). In short, musical training reflects the formal amount of musical training received, while active engagement reflects the time and effort spent on music-related activities.

Each item was scored on a seven-point Likert scale (1: “absolutely disagree” to 7: “absolutely agree”), with higher mean scores reflecting higher levels of active engagement, musical training, and musical anhedonia. Mean subscale scores are calculated using the sum of the values and are divided by the total number of questions for that subscale. Other more quantitative questions were scored between one through seven relating to different value categories quantifying years or hours of music training or daily listening (e.g., “I have had _ years of formal training on a musical instrument (including voice) during my lifetime”, “I can play _ musical instruments”, “I listen attentively to music for 0–15 min/15–30 min/30–60 min/60–90 min/2 h/2–3 h/4 h or more per day”). Responses were entered into the Gold-MSI scoring template to analyze the data obtained by the participants (https://shiny.gold-msi.org/gmsiscorer/).

### Definition of groups

We used the musical training subscale of the Gold-MSI to group participants into non-musician and amateur-musicians categories. Participants were categorized as amateur-musicians if they self-identified as a musician and had previously received compliments for their talents as musical performers. In addition, each participant had at least between 2 and 3 years of regular, daily practice of a musical instrument (including voice), practiced between 1 and 1.5 h or more per day on primary instrument at peak of interest, had between 1 and 2 years or more of formal training in music theory, 2 years or more of formal training on a musical instrument (including voice), and could play at least two instruments. The other participants (i.e., participants who did not meet the above requirements) were categorized as non-musicians. (see Suppl. Mat.).

### Musical anhedonia

An additional computed score reflecting musical pleasure, namely Musical Anhedonia (MA), was also calculated using the Gold-MSI configurator (https://shiny.gold-msi.org/gmsiconfigurator/) which selected questions from the Gold-MSI, and modeled after the Barcelona Music Reward Questionnaire (Mas-Herrero et al. 2013). The MA subscale is defined as the level of musical pleasure that is experienced when participating in music-related activities, in addition to the emotional valence engendered from music. Higher scores of MA reflect lower levels of musical anhedonia and thus show sensitivity to musical pleasure, while low MA scores reflect higher levels of musical anhedonia, reflecting insensitivity to musical pleasure. Reliability statistics were performed and Cronbach’s alpha reliability was found to be 0.813 for seven total items. For calculation of the MA subscale complete with questions used and Cronbach’s alpha reliability scores, see Suppl. Mat.

### Resilience questionnaire

We administered the 15-item Norwegian version of the Dispositional Resilience Scale (DRS-15, Hystad et al. 2010) to assess “hardiness”. Hardiness is defined as a grouping of personality characteristics that function as a resilience resource when encountering stressful life events (Kobasa 1979; Kobasa et al. 1982). “Persons high in hardiness involve themselves in whatever they are doing (commitment), believe and act as if they can influence events forming their lives (control), and consider change to be not only normal but also a stimulus to development (challenge)” (Kobasa et al. 1983). The DRS-15 includes three subscales: *commitment, control,* and *challenge.*

### Brain structure

#### MRI acquisition

For all participants, MR data were acquired at the Haukeland University Hospital in Bergen, Norway. Neuroimaging was performed on a 3.0 Tesla (T) GE Discovery MR 750 scanner (General Electric Medical Systems, Milwaukee, WI, United States) using a 32-channel head coil. T1-weighted anatomical scans were acquired using Sagittal 3D T1-weighted fast spoiled gradient-echo (FSPGR) sequence [repetition time/echo time (TR/TE) = 6.9/3.0 ms; slice thickness = 1.0 mm^3^; field of view = 25.6 cm; 256 by 256 matrix; flip angle = 12°, inversion time (TI) = 450 ms. Participants were shown nature landscapes and chose to listen to instrumental music of their choice between six music genres (e.g., pop, rock, folk, jazz, classical, and world) for a total of 9 min.

#### MRI data preprocessing and data reduction

All MR images were converted from Dicom to Nifti files using dcm2niix (Chris Rorden, version v1.0.20170724, https://www.nitrc.org/projects/mricrogl/) in MRIcroGL and then defaced using mri_deface (https://surfer.nmr.mgh.harvard.edu/fswiki/mri_deface) in FreeSurfer version 6.0.0 (https://surfer.nmr.mgh.harvard.edu/). Preprocessing of T1-weighted images in SPM12 (http://www.fil.ion.ucl.ac.uk/spm) and the CAT12 (http://dbm.neuro.uni-jena.de) toolbox (Gaser et al. 2022) in Matlab R2021b (The MathWorks Inc., Natick, MA, USA). All T1-weighted images were corrected for bias-field inhomogeneities (Van Leemput et al. 1999; Cohen et al. 2000) and spatially normalized. The images were then segmented into gray matter (GM), white matter (WM) and CSF (Ashburner and Friston 2005) which also included an approach accounting for partial volume effects (Tohka et al. 2004) by applying adaptive maximum a posteriori estimations (Rajapakse et al. 1997)⁠ and a hidden Markov Random Field Model (Cuadra et al. 2005) which has been described in (Franke et al. 2010). GM maps were registered using an affine registration and smoothed with a 4 mm and 8 mm full-width-at-half-maximum kernel and further resampled to a spatial resolution of 4 mm and 8 mm. Next, since neighboring voxels are spatially correlated and, therefore, contain redundant information, principal component analysis (PCA) was conducted to reduce data dimensions using the “Matlab toolbox for Dimensionality Reduction” (https://lvdmaaten.github.io/drtoolbox/).

#### BrainAGE framework

The BrainAGE framework is based on relevance vector regression (Tipping 2001) that transforms training data into a high-dimensional space (Bennett and Campbell 2000) and then further translates features learned from a training sample for an outcome variable (e.g., age) onto an unknown test sample.

For training, we used 1094 healthy subjects (480 males/614 females, mean age/SD 53.1 ± 21.2 years, range 18–94 years) from several databases: IXI (https://brain-development.org/ixi-dataset/), ADNI (https://adni.loni.usc.edu/) and OASIS (https://www.oasis-brains.org/). The 8 different models (4 mm and 8 mm for resampling and smoothing, gray and white matter) were combined to one single BrainAGE value using a general linear model that maximized BrainAGE differences between all groups. The difference between the estimated age and the chronological age yields the so-called brain age gap estimate (BrainAGE).

Positive BrainAGE scores (years/months) reflect accelerated aging whereby the estimated age is higher than the chronological age. Negative BrainAGE scores reflect the opposite, reflecting decelerated aging where the estimated age is lower than the chronological age. For example, a BrainAGE score of + 3.0 of an individual with a chronological age of 40 would present as having a brain age of 43 years. Lastly, a correction was made for a quadratic age trend and was applied using spm_detrend (SPM12, The Wellcome Dept. of Imaging Neuroscience, London; www.fil.ion.ucl.ac.uk/spm).

#### Performance of the BrainAGE model for brain aging from early to late adulthood

In a previous brain aging study, it was shown that, in terms of the performance of the BrainAGE model for brain aging from early into late adulthood, the measure of accuracy of age estimation resulted in a mean absolute error (MAE) of approximately 5 years and an overall correlation of *r* = 0.92, with a 95% confidence interval for the prediction of age being stable across age range (Franke et al. 2010).

### Statistical analyses

All statistical analyses were carried out using SPSS software (IBM Corp. Released 2020. IBM SPSS Statistics for Windows, Version 27.0. Armonk, NY: IBM Corp) and JASP (JASP Team (2022). JASP (Version 0.16.3). Addressing our main hypotheses, to identify possible predictors of BrainAGE scores, a forward stepwise linear regression was computed with BrainAGE scores as dependent variable, and the following independent variables: musical training, active engagement, and years of education, as well as gender as factor (years of education and gender were added in order to control for a possible influence of these variables; for additional analyses see Suppl Mat.). In addition, BrainAGE scores were compared between the groups of non-musicians and amateur-musicians using analysis of covariance (ANCOVA) and independent samples *t-*tests. Shapiro–Wilk tests for normality were performed for all *t-*test comparisons. All ANCOVA contrasts were performed using gender as a fixed factor and years of education as covariate. Since some of the items on the Gold-MSI were not normally distributed, such as “I do not consider myself a musician”, “years of training” or “years of music theory”, tests of normality were performed on the Gold-MSI subscales using Kolmogorov–Smirnov tests. Therefore, any correlation analyses using the Gold-MSI subscales were performed using non-parametric correlations (using Spearman’s rho (*r*_*s*_)).

## Results

### Musical training, active musical engagement and BrainAGE scores

To investigate whether musical training or active engagement in music predict BrainAGE scores, we computed a stepwise linear regression (with BrainAGE scores as dependent variable and MT scores, AE scores, and years of education as independent variables). This regression did not indicate a significant result (*R*^2^ = 0.01, *p* = 0.81; for complete statistics see Suppl. Mat.). Thus, our hypothesis that a background of musical training or active engagement in music would influence BrainAGE scores was rejected.

### Musician status and BrainAGE scores

**Fig. 1:**
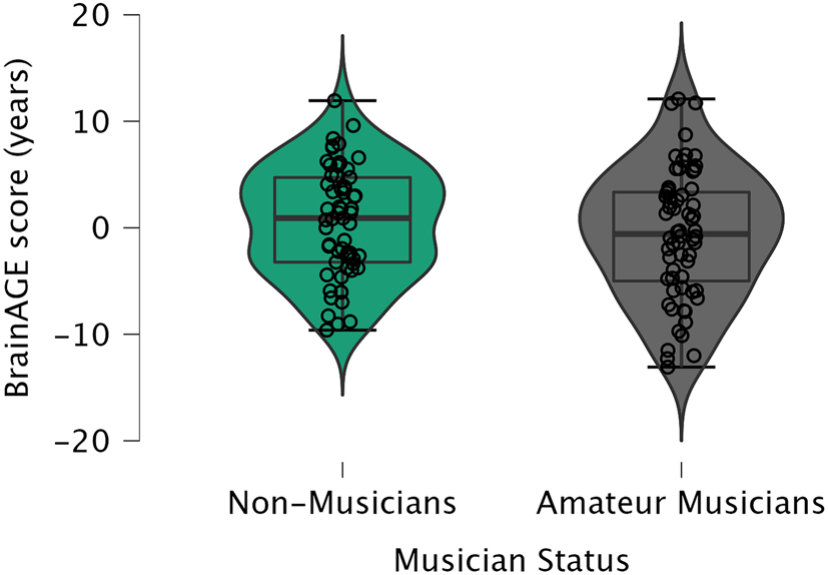
Distribution of the BrainAGE scores, separately for non-musicians and amateur-musicians. Each circle represents the BrainAGE score of an individual (in years, indicating the gap between chronological and estimated brain age). Positive scores reflect an age-accelerating brain effect (i.e., the age of the brain is older than the actual age), while negative scores reflect an age-decelerating brain effect

In addition, to provide a comparison with the previous BrainAGE study by Rogenmoser et al. (2018), we also computed a *t*-test comparing the BrainAGE scores between non-musicians (*M* = 0.56, *SD* = 5.14*, N* = 61) and amateur-musicians (*M* = − 0.65, *SD* = 6.07, *N* = 64). This *t*-test did not yield a significant result (*t*(123) = 1.20, *p* = 0.23, two-tailed). (For additional statistics, see Suppl. Mat.). These results are consistent with the stepwise linear regression indicating that BrainAGE scores are not impacted by a background of musical training, nor musical engagement. Mean Gold-MSI and musical anhedonia scores for non-musician and amateur-musicians are listed in Table 2 (Fig. 1).

**Table 2:** Mean Gold-MSI and musical anhedonia scores for non-musicians and amateur-musicians

Baseline characteristics	Non-musicians (*N* = 61)	Amateur-musicians (*N* = 64)
Musical training	1.94 (0.62)	4.67 (1.11)
Active engagement	3.43 (1.10)	4.52 (1.15)
Musical anhedonia	4.22 (0.99)	5.17 (1.09)

Listed are the means and standard deviations ((SD) reported in brackets) of the Gold-MSI subscales (MT and AE) and computed subscale MA for non-musicians and amateur-musicians

### Musical anhedonia and BrainAGE scores

To investigate whether musical anhedonia predicts BrainAGE scores, we computed a stepwise linear regression (with BrainAGE scores as dependent variable, independent variables: musical anhedonia scores and years of education). This regression did not indicate a significant result (*R*^2^ = 0.04, *p* = 0.89). (For additional statistics including direct comparisons between non-musicians and amateur-musicians, see Suppl. Mat.).

### Exploratory analyses

#### Music and resilience

We performed Spearman’s rank correlations investigating the relationship between AE, MT, and MA on the DRS-15 subscales (control, challenge, and commitment) and found a significant correlation to the subscale of challenge (Spearman’s correlation coefficients can be found in Table 3 and Pearson’s *r* correlation coefficients can be found in Suppl. Mat., along with additional statistical analyses).

**Table 3:** Spearman’s rank correlation matrix

	Resilience total	Commitment	Control	Challenge
Active engagement	*r*_*s*_ = 0.166, *p* = 0.06	*r*_*s*_ = 0.144, *p* = 0.11	*r*_*s*_ = 0.039, *p* = 0.67	*r*_*s*_ = 0.185, *p* = 0.04*
Music training	*r*_*s*_ = 0.147, *p* = 0.10	*r*_*s*_ = 0.084, *p* = 0.35	*r*_*s*_ = −0.005, *p* = 0.95	*r*_*s*_ = 0.214, *p* = 0.02*
Musical anhedonia	*r*_*s*_ = 0.162, *p* = 0.07	*r*_*s*_ = 0.075, *p* = 0.40	*r*_*s*_ = 0.088, *p* = 0.33	*r*_*s*_ = 0.216, *p* = 0.02*

*Correlation is significant at the 0.05 level (2-tailed)

#### Resilience and BrainAGE scores

To explore whether resilience predicts BrainAGE scores, we computed a stepwise linear regression (with BrainAGE scores as dependent variable and resilience scores and years of education as independent variables). The regression analysis did not indicate that resilience predicted BrainAGE scores (*R*^2^ = 0.01, *F*(2, 122) = 0.35, *p* = 0.71). (For additional statistics including direct comparisons between non-musicians and amateur-musicians, see Suppl. Mat.).

#### Lifestyle factors and BrainAGE scores

Exploratory analyses were performed to generate hypotheses for future studies; therefore, results will be minimally discussed. Firstly, we explored whether those who were currently engaged in physical activity had significantly different BrainAGE scores than those who were not physically active.

An independent samples *t-*test examining lifestyle factors like self-reported current physical activity against BrainAGE scores revealed that healthy adults who were currently engaged in physical activity had lower BrainAGE scores (*M* = −0.63, *SD* = 5.75*, N* = 97) than those who were not physically active (*M* = 1.95, *SD* = 4.85*, N* = 28), *t*(123) = 2.17, *p* = 0.03, two-tailed. (See Suppl. Mat, for violin plots and complete statistics).

A further independent samples *t*-test was performed examining the self-reported use of allergy medication in comparison to BrainAGE scores. Results showed that individuals who used allergy medication had lower BrainAGE scores (*M* =−3.71, *SD* = 4.24, *N* = 10) versus those who did not (*M* = 0.26, *SD* = 5.66, *N* = 115), *t*(123) = 2.17, *p* = 0.03, two-tailed. (See Suppl. Mat. for complete statistics).

## Discussion

### Musician status on BrainAGE

The present study investigates the relationship between aspects of musical sophistication as measured by two subscales of the Gold-MSI self-inventory of musical sophistication index (Goldsmiths Musical Sophistication Index; Gold-MSI; (Müllensiefen et al. 2014), namely active engagement and musical training, and a brain aging metric as measured by BrainAGE (Franke et al. 2010). The study failed to replicate the findings from a previous BrainAGE and music-making study (Rogenmoser et al. 2018) revealing no association between musician status and BrainAGE scores. Whether participants had previous musical training or were currently actively engaged in music, the brain age scores were not significantly affected. However, we found a slight (non-significant) trend suggesting more negative brain age scores among amateur-musicians (*M* = −0.65) compared to (*M* = 0.56) non-musicians, reflecting a possible age-decelerating effect of musical training on brain age scores. However, certain sample differences must be outlined between our study and the Rogenmoser et al. (2018) study. The ages of their sample ranged between 17 and 39 years of age, with means of each group around 25 years of age. In contrast, our sample ranged between 22 to 89 years of age, with non-musicians and amateur-musicians having mean ages of 54 and 51 years, respectively. Our sample selection may have neutralized potential effects of musician status on BrainAGE scores because those with a musical background may have spent many years without concurrent practice. However, a strength of the current study is that the large age range may allow for better quantification of musical training effects for normative aging across the lifespan, and points to the potential beneficial effects of continuous musical practice. Potential confounding factors such as ‘age of onset’ of musical training and details providing training intensity levels should be considered in future studies.

### Goldsmiths musical sophistication index: musical training and active engagement on BrainAGE

We measured two subscales from the well-validated Gold-MSI questionnaire which reflects musicality and musical sophistication (Müllensiefen et al. 2014), (Fiedler and Müllensiefen 2015) in non-professional musician cohorts. We found that neither MT nor AE were correlated to BrainAGE score. Both the MT and AE subscales are closely associated with motor and somatosensory activities (Müllensiefen et al. 2014), for example, both musical training and active musical engagement are directly related to auditory and motor systems, regardless of the specific activities or instruments that are used. As the BrainAGE metric takes into account whole brain volumetric data, it may not be an ideal metric to investigate the association between musicality and brain aging. Defining areas in the gray and white matter relating specifically to motor, somatosensory, and auditory areas may be preferable in future studies. For example, a study investigating sex differences in white matter microstructural alterations in regards to MT and AE of the Gold-MSI found that both aspects of musical sophistication were correlated to several white matter tracts relating to motor and somatosensory functions, finding males had more significant white matter integrity in these areas versus females in their sample (Mehrabinejad et al. 2021). Outlining either white matter tracts of interest using diffusion MRI, or regions of interest using volumetric analysis of gray matter, that are directly related to motor, somatosensory, and auditory functions may be of interest for future work.

Further, based on a general consensus by music psychologists, “musician” status can be based on years of musical training for a period of at least 6 years (Zhang et al. 2020). The MT subscale can capture this data, however regardless of the amount of musical training in our sample, we did not see any associations with BrainAGE score. Since little is known on how musical training affects gray matter structure in older adults (Chaddock-Heyman et al. 2021), BrainAGE estimates can reflect the general health of brain, including gray matter volume. In our study, we tended to be more conservative and although some individuals had musical training over 6 years, we still aimed to group “amateur-musicians” if they met certain criteria using the items on the MT subscale. We toggled the boundary between non-musician and amateur-musician groups, finding that despite levels of musical training and active musical engagement, both did not predict BrainAGE scores (see Suppl. Mat.). It may be advantageous to include ‘age of onset’ of musical training, intensity of musical training, a subgroup of professional musicians, and the addition of other tests of musicality in future studies for better quantification of effects.

### Lifestyle and physical activity on BrainAGE

Although the study did not show any significant associations of musical training and active musical engagement to BrainAGE scores, exploratory analyses suggest that healthy adults who were currently physically active had significantly different BrainAGE scores than those who were not currently active. A previous study (Bittner et al. 2021) that examined the brains of 622 older adults found physical activity to be predictive for BrainAGE in males (*p* = 0.005), but not in females. Controlling for gender, and years of education, our findings suggest that current involvement in physical activity was found to be predictive of BrainAGE scores (*p* = 0.02) scores for both genders.

Our study also found significantly different BrainAGE scores in individuals taking allergy medication compared to those who did not. These results are consistent with similar studies finding short-term fluctuations in BAG with regards to menstruation and the use of non-steroidal anti-inflammatory drugs like ibuprofen (Franke et al. 2015; Le et al. 2018). We found that the BAG scores from individuals taking allergy medication (*M* = −3.71) compared to those who did not (*M* = 0.26), showed a significant effect of medication on BrainAGE score when controlling for gender, and years of education. Therefore, future studies may want to verify the use of: drug type, drug dosage, duration of use, and most recent use of pharmacological substances before brain scanning, as well as menstrual cycle data. These findings reveal how unexplained variance due to lifestyle factors, drug use, and menstrual cycles may have an influence on BAG values, illustrating that BAG is a dynamic process which can be influenced by a multiplicity of variables.

### Resilience, cognitive reserve, and Gold-MSI

Interestingly, another incidental finding revealed that musical training, active musical engagement, and musical anhedonia were significantly correlated to dispositional resilience scores, namely resilience to challenge, as measured by the DRS-15 (Hystad et al. 2010), but not to BrainAGE scores. As this analysis was meant only to generate hypotheses for future studies, preliminary results indicate that resilience and music may be correlated and perhaps the concept of cognitive “reserve” may explain the robustness of these findings suggesting that music-making may be associated with building resilience.

Resilience may be defined as the ability of an individual to maintain stable psychological functioning throughout the course of adversity (Marley and Mauki 2019). Building resilience requires the development of internal factors such as self-control, emotional regulation, self-esteem, agency, and certain external factors such as the development of social skills, connections, and close relationships which can both serve as internal and external protective factors (Barankin and Khanlou 2007). Resilience, which can be both defined as a personality trait and as an adaptive process that can be developed (Harris 2008; Ong et al. 2009; MacLeod et al. 2016), can also be analogous to a neurocognitive metric for longevity as studies have shown that adults aged 85 and older seem to have the same or even greater capacity for resilience as younger people (Gooding et al. 2012; Netuveli et al. 2008).

The concept of reserve is explained via the individual variability in the functional use or structural integrity of the nervous system that modifies cognitive and behavioral abilities following the onset of brain pathology or general biological aging. BrainAGE scores are one example of a marker of brain aging that can be used as a potential metric for “brain reserve”. In contrast, cognitive reserve is regarded as an “active” mechanism for coping with brain pathology. This may outline why BrainAGE scores were not related to musical training nor active engagement in music.

In contrast, our findings suggest that active engagement, musical training, and musical anhedonia were related to resilience to challenge. Perhaps both the domain of adaptation to challenge and musical proficiency are related to the process of learning and potentially how the successful adaptation to stress may widen the capacity for cognitive flexibility (McEwen et al. 2015). In a review on resilience building through music and motion, Nijs and Nicolaou (2021) suggest how music-making and resilience are intimately connected. Not only can music provide pleasure and meaning (Lamont 2011), but it can also contribute to social relatedness which can aid in the expression of one’s identity and values, all while forging connection with others (Schäfer et al. 2013). The active engagement with music, particularly in group settings (e.g., choirs), contributes both internal and external protective factors of resilience which is known to support longevity (MacLeod et al. 2016).

### Limitations and future directions

Health and disease are not binary classifications but the result of complex interactions between various factors over long time spans. Most brain aging studies have been exclusively correlational, and findings describing the effects of lifestyle factors on brain age have added complexity in how the concept of brain aging and the use of such metrics, such as the brain age gap, BAG, are interpreted. In addition, studies analyzing menstrual cycle and the use of NSAID drugs (Franke et al. 2015),(Le et al. 2018) showcase that BrainAGE estimation can be temporarily affected, either increased or decreased, and thus natural fluctuations based on certain drugs or lifestyle factors must be taken into consideration.

The predictive accuracy of brain aging algorithms has also been called into question. The predictive accuracy of brain aging algorithms differ between each model and the model performance can be evaluated with several metrics such as R^2^, root mean square error, or mean absolute error between real and predicted estimates (MacDonald and Pike 2021). BrainAGE has an MAE approximately between 4.5 and 5 years (Franke and Gaser 2019), in contrast to other algorithms citing MAE values of 4.16 years (Cole et al. 2017), and even 0.63 years (Beheshti et al. 2019, 2021) using patch-wise analysis. Therefore, other algorithms may provide better predictive accuracy and may be preferred in future studies.

A further limitation of our study was that we did not account for ‘age of onset’ of musical training, nor collected specific data on training intensity levels. Age of onset is considered the sensitive period whereby the strongest effects on brain and behavior can be determined. Age of onset prior to the age of seven is one of the significant predictors of specific training-induced structural brain changes (Bailey and Penhune 2013). Further, training intensity was only partially assessed via the Gold-MSI MT subscale, which can further be expounded upon using additional questionnaires in future studies.

Lastly, although participants were excluded for serious previous illness, no screening of subjective memory complaints was performed. As dementia, specifically Alzheimer’s dementia begins decades before the presentation of clinical symptoms (Rajan et al. 2015), the participants were not screened for memory problems, and could potentially be less “healthy” than is claimed. Therefore, future studies could add additional questionnaires such as the Subjective Cognitive Decline Questionnaire (SCD-Q) (Rami et al. 2014), as a screening measure to ensure the sample is cognitively healthy.

## Conclusion

This study investigated the effects of musical training, active musical engagement, musical anhedonia, and dispositional resilience on brain aging, specifically on BrainAGE scores. Although brain age scores were not related to musician status, aspects of musicality (MT and AE), nor resilience, incidental findings revealed that current involvement in physical activity and the use of allergy medication were predictive of BrainAGE scores. An exploratory analysis also showed that musician status, MT and AE, and sensitivity to musical pleasure were related to the dispositional resilience scale, DRS-15, particularly the challenge subscale. This may reflect the benefits of music-related behaviors which may contribute to build resilience over time, yet future studies are needed to investigate this potential relationship.

## Supplementary Information

Below is the link to the electronic supplementary material.

Supplementary file1 (DOCX 375 KB)

## Data Availability

The data that support the findings of this study are available from the corresponding author, A.M.M., upon reasonable request.
