# Supplementary material for: Is musical engagement enough to keep the brain young?
Source: Brain Struct Funct. 2022 Dec 27;228(2):577–88. doi: 10.1007/s00429-022-02602-x (PMC9945036; doi:10.1007/s00429-022-02602-x)
Supplement: Supplementary file 1 — Supplementary file1 (DOCX 375 KB) [file 429_2022_2602_MOESM1_ESM.docx]

**Supplementary Material**

**1. Goldsmith-Musical Sophistical Index, selected questions, 19 in total used for study**

Question 1: I spend a lot of my free time doing music-related activities.
Reversed Negative Items: 5, 6, 9 and 11.

**Scorer**: https://shiny.gold-msi.org/gmsiscorer/

**Questions about your musical background**

| **Please circle the most appropriate category:** | **1**  **Completely dis-agree** | **2**  **Strongly dis-agree** | **3**  **Dis-agree** | **4**  **Neither agree nor dis-agree** | **5**  **Agree** | **6**  **Strongly agree** | **7**  **Completely agree** |
| --- | --- | --- | --- | --- | --- | --- | --- |
| I spend a lot of my free time doing music-related activities. | **1** | **2** | **3** | **4** | **5** | **6** | **7** |
| I sometimes choose music that can trigger shivers down my spine. | **1** | **2** | **3** | **4** | **5** | **6** | **7** |
| I enjoy writing about music, for example on blogs and forums. | **1** | **2** | **3** | **4** | **5** | **6** | **7** |
| I’m intrigued by musical styles I’m not familiar with and want to find out more. | **1** | **2** | **3** | **4** | **5** | **6** | **7** |
| Pieces of music rarely evoke emotions for me. | **1** | **2** | **3** | **4** | **5** | **6** | **7** |
| I have never been complimented for my talents as a musical performer. | **1** | **2** | **3** | **4** | **5** | **6** | **7** |
| I often read or search the internet for things related to music. | **1** | **2** | **3** | **4** | **5** | **6** | **7** |
| I often pick certain music to motivate or excite me. | **1** | **2** | **3** | **4** | **5** | **6** | **7** |
| I don’t spend much of my disposable income on music. | **1** | **2** | **3** | **4** | **5** | **6** | **7** |
| Music is kind of an addiction for me - I couldn’t live without it. | **1** | **2** | **3** | **4** | **5** | **6** | **7** |
| I would not consider myself a musician. | **1** | **2** | **3** | **4** | **5** | **6** | **7** |
| I keep track of new music that I come across (e.g. new artists or recordings). | **1** | **2** | **3** | **4** | **5** | **6** | **7** |
| I engaged in regular, daily practice of a musical instrument (including voice) for … years. | **0** | **1** | **2** | **3** | **4-5** | **6-9** | **> 9** |
| At the peak of my interest, I practiced … hours per day on my primary instrument. | **0** | **0,5** | **1** | **1,5** | **2** | **3-4** | **> 4** |
| I have attended ... live music events as an audience member in the past twelve months. | **0** | **1** | **2** | **3** | **4-6** | **7-10** | **> 10** |
| I have had formal training in music theory for … years. | **0** | **0,5** | **1** | **2** | **3** | **4-6** | **> 6** |
| I have had … years of formal training on a musical instrument (including voice) during my lifetime. | **0** | **0,5** | **1** | **2** | **3-5** | **6-9** | **> 9** |
| I can play ... musical instruments. | **0** | **1** | **2** | **3** | **4** | **5** | **> 5** |
| I listen attentively to music for … per day. | **0-15 min** | **15-30 min** | **30-60 min** | **60-90 min** | **2 hrs** | **2-3 hrs** | **> 3 hrs** |

**2. Calculation of Groups from MT score**

**2.1** To be categorized as an amateur-musician, each participant had at least neutral or answered in the affirmative when asked if they consider themselves musicians and had received compliments for their talents as a musical performer, had at least 3 years of regular, daily practice of a musical instrument (including voice), practiced at least 1.5 hours or more per day on primary instrument at peak of interest, had at least 2 years or more of formal training in music theory, at least 2 years or more of formal training on a musical instrument (including voice), and could play at least 3 instruments or more. Non-musicians were considered to have less than the above requirements. This meant that non-musicians had a mean MT score below 4/7 and amateur-musicians had a score above 4.

Syntax in SPSS: T-TEST GROUPS=LowBelow4_VsOver4(0 1)

/MISSING=ANALYSIS

/VARIABLES=BrainAge2022

*/CRITERIA=CI(.95).*

| **Group Statistics** | | | | | |
| --- | --- | --- | --- | --- | --- |
|  | LowBelow4_VsOver4 | N | Mean | Std. Deviation | Std. Error Mean |
| BrainAge2022 | .00 | 77 | .1572 | 5.40830 | .61633 |
|  | 1.00 | 48 | -.3957 | 6.05319 | .87370 |

Shapiro-Wilk test showed both are equal in variance (between 77 (low) and 48 (high) MT groups)

Independent samples t-test: *t*(123)= 0.531 *p =* 0.596, 2-tailed.

**2.2** We also divided the groups in three, non-musician, amateur, and musician, yet they still did not have any significant effect on brainAGE score. In this case, musician was selected for participants who had at least 6 to 9 years of practice on an instrument, at least 4 to 6 years of formal music theory, and music training, and strongly agreed that they considered themselves musicians and were praised on their musical skill.

Syntax in SPSS: ONEWAY BrainAGE BY Musician_Status_Non_vs_Amateur_vs_Musician

/MISSING ANALYSIS.

| **Report** | | | |
| --- | --- | --- | --- |
| BrainAGE | | | |
| Musician_Status_Non_vs_Amateur_vs_Musician | Mean | N | Std. Deviation |
| Non Musician | .60166129 | 62 | 5.106127284 |
| Amateur Musician | -.93527451 | 51 | 6.082850307 |
| Musician | .29232500 | 12 | 6.367286671 |
| Total | -.05510480 | 125 | 5.646969729 |

| **ANOVA** | | | | | |
| --- | --- | --- | --- | --- | --- |
| BrainAGE | | | | | |
|  | Sum of Squares | df | Mean Square | F | Sig. |
| Between Groups | 67.701 | 2 | 33.851 | 1.063 | .349 |
| Within Groups | 3886.444 | 122 | 31.856 |  |  |
| Total | 3954.145 | 124 |  |  |  |

**3. Calculation of Musical Anhedonia, MA, subscale in SPSS**

*ANHEDONIA - BMRQscale.
COMPUTE MUS_ANHEDONIA=(MS02_01 + MS02_03 + MS02_04_r + MS02_06 + MS02_07
+ MS02_08_r + MS02_09)/7.
EXECUTE.

VARIABLE LABELS MS02_01 'Spend a lot of free time doing music related
activities'.
ADD VALUE LABELS MS02_01 '1' 'completely disagree' '2' 'strongly
disagree' '3' 'disagree' '4' 'neither agree nor disagree' '5' 'agree'
'6' 'strongly agree' '7' 'completely agree' '-9' 'not answered'.
VARIABLE LEVEL MS02_01 (SCALE).
ALTER TYPE MS02_01 (f2).

VARIABLE LABELS MS02_03 'often read or search internet for music'.
ADD VALUE LABELS MS02_03 '1' 'completely disagree' '2' 'strongly
disagree' '3' 'disagree' '4' 'neither agree nor disagree' '5' 'agree'
'6' 'strongly agree' '7' 'completely agree' '-9' 'not answered'.
VARIABLE LEVEL MS02_03 (SCALE).

VARIABLE LABELS MS02_04 'do NOT spend much of income on music'.
ADD VALUE LABELS MS02_04 '1' 'completely disagree' '2' 'strongly
disagree' '3' 'disagree' '4' 'neither agree nor disagree' '5' 'agree'
'6' 'strongly agree' '7' 'completely agree' '-9' 'not answered'.
VARIABLE LEVEL MS02_04 (SCALE).
RECODE MS02_04 (1=7) (2=6) (3=5) (4=4) (5=3) (6=2) (7=1) INTO MS02_04_r.
VARIABLE LABELS  MS02_04_r 'Reversert'.
EXECUTE.

VARIABLE LABELS MS02_06 'I keep track of new music'.
ADD VALUE LABELS MS02_06 '1' 'completely disagree' '2' 'strongly
disagree' '3' 'disagree' '4' 'neither agree nor disagree' '5' 'agree'
'6' 'strongly agree' '7' 'completely agree' '-9' 'not answered'.
VARIABLE LEVEL MS02_06 (SCALE).

VARIABLE LABELS MS02_07 'choose music that can trigger shivers down my
spine'.
ADD VALUE LABELS MS02_07 '1' 'completely disagree' '2' 'strongly
disagree' '3' 'disagree' '4' 'neither agree nor disagree' '5' 'agree'
'6' 'strongly agree' '7' 'completely agree' '-9' 'not answered'.
VARIABLE LEVEL MS02_07 (SCALE).

VARIABLE LABELS MS02_08 'piecec of music RARELY evoke emotions for me'.
ADD VALUE LABELS MS02_08 '1' 'completely disagree' '2' 'strongly
disagree' '3' 'disagree' '4' 'neither agree nor disagree' '5' 'agree'
'6' 'strongly agree' '7' 'completely agree' '-9' 'not answered'.
VARIABLE LEVEL MS02_08 (SCALE).
RECODE MS02_08 (1=7) (2=6) (3=5) (4=4) (5=3) (6=2) (7=1) INTO MS02_08_r.
VARIABLE LABELS  MS02_08_r 'Reversert'.
EXECUTE.

VARIABLE LABELS MS02_09 'often pick certain music to motivate or excite me'.
ADD VALUE LABELS MS02_09 '1' 'completely disagree' '2' 'strongly
disagree' '3' 'disagree' '4' 'neither agree nor disagree' '5' 'agree'
'6' 'strongly agree' '7' 'completely agree' '-9' 'not answered'.
VARIABLE LEVEL MS02_09 (SCALE).

RECODE
(-9 = SYSMIS).
EXECUTE.
* Compute Cronbach's alpha.
RELIABILITY /VARIABLES= MUS_ANHEDONIA
ALL /MODEL=ALPHA /STATISTICS=SCALE.

| **Reliability Statistics** | |
| --- | --- |
| Cronbach's Alpha | N of Items |
| .813 | 7 |

**4.1** Relationship between chronological (biological age) in years and brain predicted age (brainAGE) in years. As shown in the scatterplot below, there is no age-depency featured in the relationship between age and BrainAGE scores.


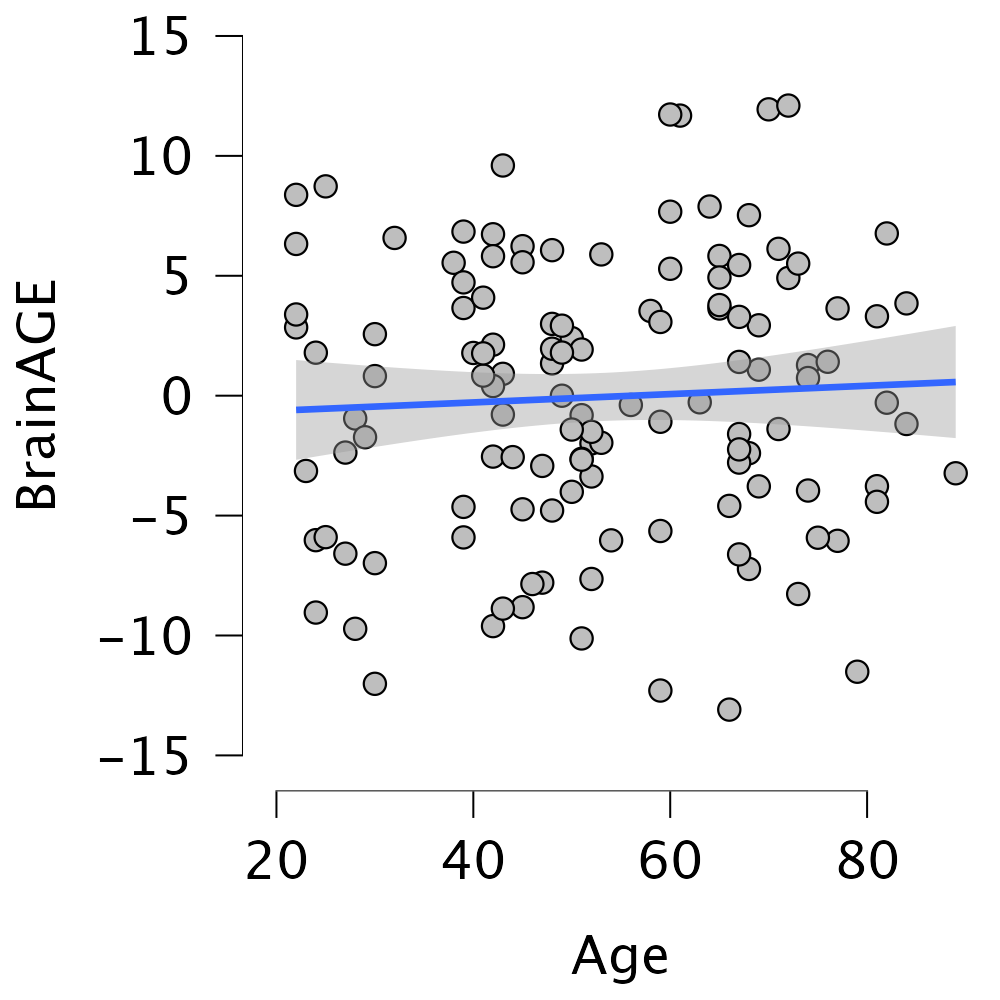


**Figure 3. Scatterplot showing the lack of age-dependency in BrainAGE scores**

4.2 Relationship between BrainAGE scores depicted in years with the Gold-MSI subscales in amateur-musicians and non-musicians.


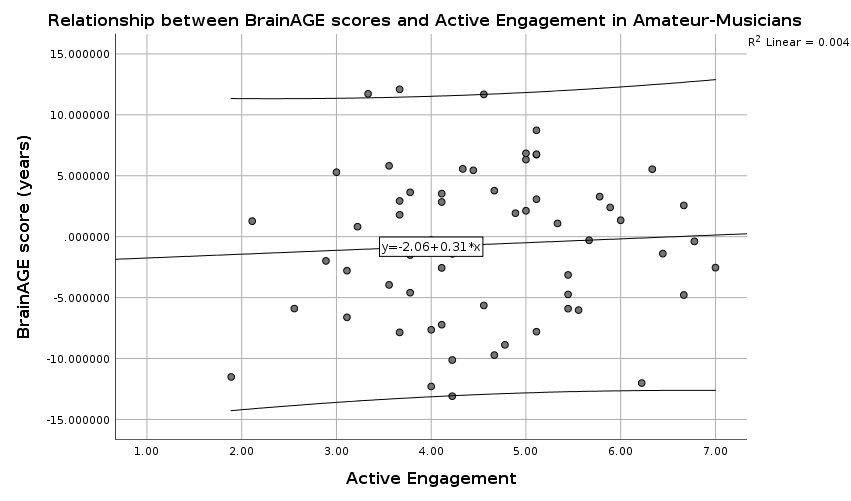


**Fig. 4** Relationship between BrainAGE scores (in years) and active engagement in amateur-musicians. Each dot depicts the individual pairs of BrainAGE scores and Gold-MSI Active Engagement subscale scores in healthy participants. The straight line indicates the best linear fits and the R^2^ indicates the explained variance, with outer lines depicting 95% confidence intervals.


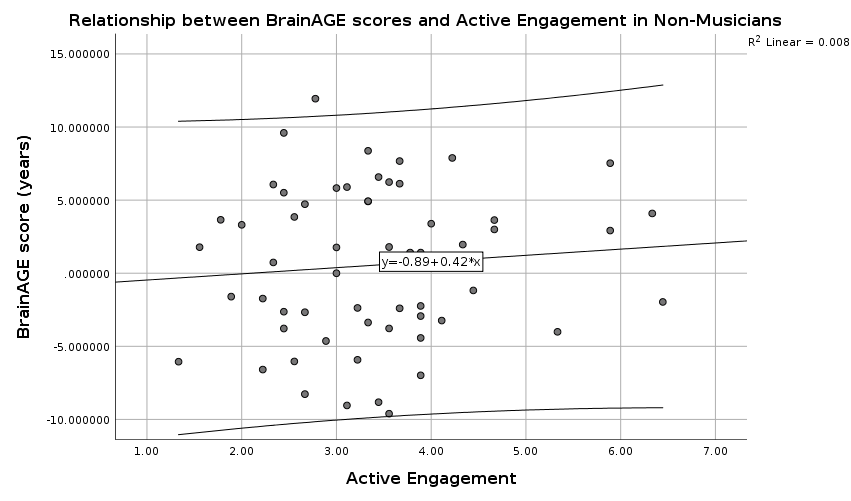


**Fig. 5** Relationship between BrainAGE scores (in years) and active engagement in non-musicians. Each dot depicts the individual pairs of BrainAGE scores and Gold-MSI Active Engagement subscale scores in healthy participants. The straight line indicates the best linear fits and the R^2^ indicates the explained variance, with outer lines depicting 95% confidence intervals.


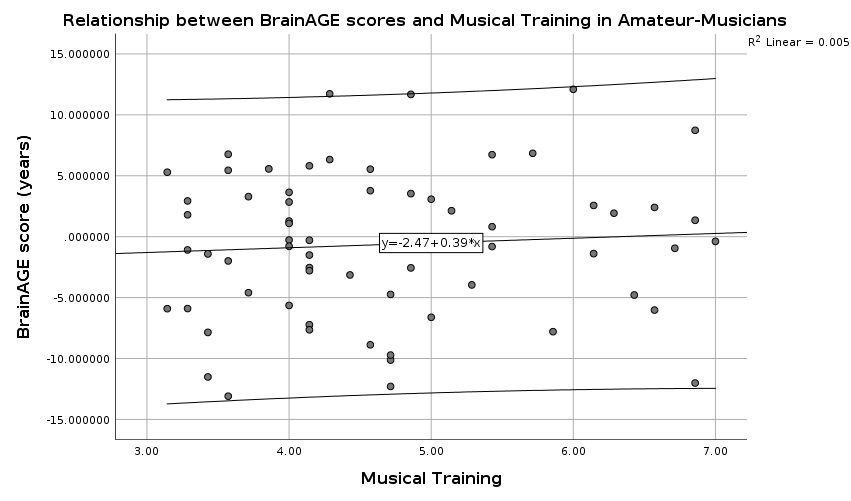


**Fig. 6** Relationship between BrainAGE scores (in years) and musical training in amateur-musicians. Each dot depicts the individual pairs of BrainAGE scores and Gold-MSI Musical Training subscale scores in healthy participants. The straight line indicates the best linear fits and the R^2^ indicates the explained variance, with outer lines depicting 95% confidence intervals.


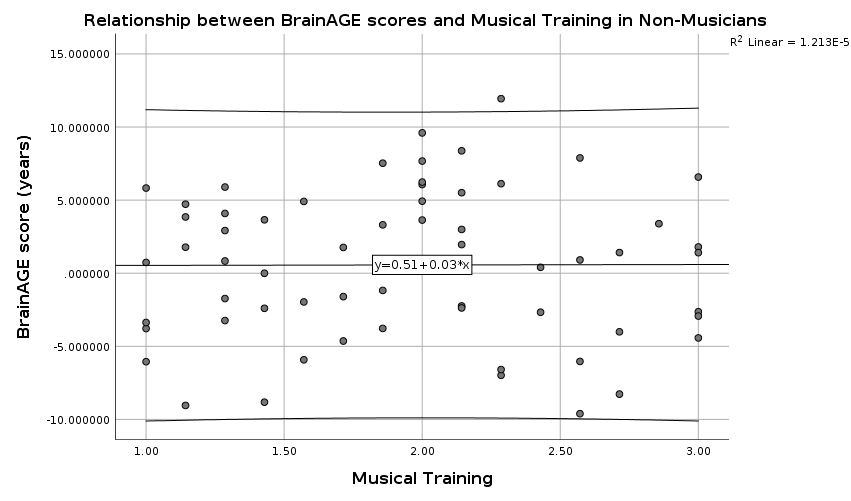


**Fig. 7** Relationship between BrainAGE scores (in years) and musical training in non-musicians. Each dot depicts the individual pairs of BrainAGE scores and Gold-MSI Musical Training subscale scores in healthy participants. The straight line indicates the best linear fits and the R^2^ indicates the explained variance, with outer lines depicting 95% confidence intervals.

**5 Results: additional computed statistics**

**5.1 Musical training, active musical engagement and BrainAGE scores**

We computed a stepwise linear regression (with BrainAGE scores as dependent variable, independent variables: MT scores, AE scores, and years of education), *R*^2^ = 0.01, *p* = 0.81, *F*(3,121) = 0.32, *β* = -0.42, 95% CI [-5.74, 4.90]. Our hypothesis that a background of musical training or active engagement in music would influence BrainAGE scores was rejected.

Non-parametric analyses using Spearman’s rank correlations were computed to assess the relationships between aspects of musicality (MT and AE), and BrainAGE scores. Both contrasts resulted in non-significant Spearman correlations between MT and BrainAGE (*rs* = -0.07, *p* = 0.45, *N* = 125), and AE and BrainAGE (*rs* = 0.03, *p* = 0.76, *N* = 125). No further correlations were found for between MA and BrainAGE (*rs* = -0.04, *p* = 0.69, *N* = 125).

**5.2 Musician status and BrainAge scores**

A direct comparison of musician status and BrainAGE scores (using ANCOVA with BrainAGE scores as dependent variable, years of education as covariate, fixed factors: musician status and gender) did not yield a significant result, *F*(1,121) = 1.44, *p* = 0.23.

**5.3 Musical anhedonia and BrainAge scores**

To investigate whether musical anhedonia predicts BrainAGE scores, we computed a stepwise linear regression (with BrainAGE scores as dependent variable, independent variables: musical anhedonia scores and years of education). This regression did not indicate a significant result, (*R*^2^ = 0.04, *p* = 0.89, *F* (2,122) = 0.11, *β* = -0.21, 95% CI [-1.12, 0.69].

Likewise, a direct comparison of musical anhedonia scores and BrainAGE scores (using an ANCOVA with BrainAGE scores as dependent variable, covariates: musical anhedonia and years of education, fixed factors: musician status and gender) did not yield a significant result, *F*(1,119) = 0.22, *p* = 0.64.

A direct comparison of musical anhedonia scores between the groups of non-musicians and amateur musicians using independent samples *t-*tests indicated that amateur-musicians (*M =* 5.17, *SD =* 1.09, *N =* 64) are significantly more sensitive to musical pleasure than non-musicians (*M =* 4.22, *SD =* 0.99*, N =* 61) in our sample, (*t*(123) = -5.10 *p =* 0.23, two-tailed). Higher MA scores reflect higher levels of musical pleasure, and lower MA scores reflect the presence of musical anhedonia, or lack of reward to musical pleasure.

**5.4 Exploratory Analyses: Resilience*,* Music, and BrainAGE scores**

**Music, Resilience, and Musical Anhedonia**

To explore whether musical training or musical engagement predict total resilience score, we computed a stepwise linear regression (with resilience scores as dependent variable, independent variables: MT scores, AE scores, years of education, and age). This regression did not indicate a significant result (*R*^2^ = 0.04, *F*(4, 120) = 1.16, *p* = 0.33).

Likewise, a direct comparison of resilience scores between the groups of non-musicians and amateur musicians (using an ANCOVA with resilience scores as dependent variable, covariates: age, and years of education, fixed factors: musician status and gender) did not yield a significant group difference for total resilience scores (*F*(1,119) = 1.70; *p* = 0.20), however it did yield a significant group difference for resilience to challenge (*F*(1,119) = 4.13; *p* = 0.04).

Spearman’s rank and Pearson’s (*r)* correlations investigating the relationship between AE, MT, and MA on Resilience (total and subscale scores) can be found in Tables 1 through 3.

**Table 1.** Correlation table for the association between Resilience and Musical Training

| Correlation Table | | | | | | | | | | | | | |
| --- | --- | --- | --- | --- | --- | --- | --- | --- | --- | --- | --- | --- | --- |
|  | | | | | | Pearson | | | | Spearman | | | |
|  | |  | |  | | r | | p | | rho | | p | |
| Resilience_Total |  | - |  | Resilience_Commitment |  | 0.6165 | *** | 1.9388e-14 |  | 0.6638 | *** | 3.2592e-17 |  |
| Resilience_Total |  | - |  | Resilience_Control |  | 0.7083 | *** | 2.5213e-20 |  | 0.6942 | *** | 2.8041e-19 |  |
| Resilience_Total |  | - |  | Resilience_Challenge |  | 0.7151 | *** | 7.4764e-21 |  | 0.6751 | *** | 5.9286e-18 |  |
| Resilience_Total |  | - |  | MusicalTraining |  | 0.1424 |  | 0.1131 |  | 0.1474 |  | 0.1008 |  |
| Resilience_Commitment |  | - |  | Resilience_Control |  | 0.1891 | * | 0.0347 |  | 0.2666 | ** | 0.0027 |  |
| Resilience_Commitment |  | - |  | Resilience_Challenge |  | 0.1443 |  | 0.1084 |  | 0.1928 | * | 0.0313 |  |
| Resilience_Commitment |  | - |  | MusicalTraining |  | 0.0709 |  | 0.4322 |  | 0.0843 |  | 0.3498 |  |
| Resilience_Control |  | - |  | Resilience_Challenge |  | 0.2527 | ** | 0.0045 |  | 0.2280 | * | 0.0105 |  |
| Resilience_Control |  | - |  | MusicalTraining |  | -0.0037 |  | 0.9669 |  | -0.0052 |  | 0.9542 |  |
| Resilience_Challenge |  | - |  | MusicalTraining |  | 0.2137 | * | 0.0167 |  | 0.2136 | * | 0.0168 |  |
|  | | | | | | | | | | | | | |
| * p < .05, ** p < .01, *** p < .001 | | | | | | | | | | | | | |

**Active Engagement and Resilience**

**Table 2.** Correlation table for the association between Resilience and Active Engagement

| Correlation Table | | | | | | | | | | | | | |
| --- | --- | --- | --- | --- | --- | --- | --- | --- | --- | --- | --- | --- | --- |
|  | | | | | | Pearson | | | | Spearman | | | |
|  | |  | |  | | r | | p | | rho | | p | |
| Resilience_Total |  | - |  | Resilience_Commitment |  | 0.6165 | *** | 1.9388e-14 |  | 0.6638 | *** | 3.2592e-17 |  |
| Resilience_Total |  | - |  | Resilience_Control |  | 0.7083 | *** | 2.5213e-20 |  | 0.6942 | *** | 2.8041e-19 |  |
| Resilience_Total |  | - |  | Resilience_Challenge |  | 0.7151 | *** | 7.4764e-21 |  | 0.6751 | *** | 5.9286e-18 |  |
| Resilience_Total |  | - |  | ActiveEngagement |  | 0.1702 |  | 0.0577 |  | 0.1663 |  | 0.0638 |  |
| Resilience_Commitment |  | - |  | Resilience_Control |  | 0.1891 | * | 0.0347 |  | 0.2666 | ** | 0.0027 |  |
| Resilience_Commitment |  | - |  | Resilience_Challenge |  | 0.1443 |  | 0.1084 |  | 0.1928 | * | 0.0313 |  |
| Resilience_Commitment |  | - |  | ActiveEngagement |  | 0.1322 |  | 0.1415 |  | 0.1438 |  | 0.1096 |  |
| Resilience_Control |  | - |  | Resilience_Challenge |  | 0.2527 | ** | 0.0045 |  | 0.2280 | * | 0.0105 |  |
| Resilience_Control |  | - |  | ActiveEngagement |  | 0.0233 |  | 0.7961 |  | 0.0390 |  | 0.6658 |  |
| Resilience_Challenge |  | - |  | ActiveEngagement |  | 0.1891 | * | 0.0347 |  | 0.1848 | * | 0.0391 |  |
|  | | | | | | | | | | | | | |
| * p < .05, ** p < .01, *** p < .001 | | | | | | | | | | | | | |

**Musical Anhedonia and Resilience**

To explore whether musical anhedonia predict total resilience score, we computed a stepwise linear regression (with resilience scores as dependent variable, independent variables: musical anhedonia, age, and years of education). The regression did not indicate a significant result (*R*^2^ = 0.03, *F*(3, 121) = 1.41, *p* = 0.24, *β* = 0.63, 95% CI [-1.8, 1.44]).

Likewise, a direct comparison of musical anhedonia between the groups of non-musicians and amateur musicians (using an ANCOVA with musical anhedonia scores as dependent variable, covariates: age, resilience total scores, and years of education, fixed factors: musician status and gender) did yield a significant group difference for musical anhedonia scores (*F*(1,118) = 22.91; *p <* 0.001).

**Musical Anhedonia and Resilience**

**Table 3.** Correlation table for the association between Resilience and Musical Anhedonia

| Correlation Table | | | | | | | | | | | | | |
| --- | --- | --- | --- | --- | --- | --- | --- | --- | --- | --- | --- | --- | --- |
|  | | | | | | Pearson | | | | Spearman | | | |
|  | |  | |  | | r | | p | | rho | | p | |
| Resilience_Total |  | - |  | Resilience_Commitment |  | 0.6165 | *** | 1.9388e-14 |  | 0.6638 | *** | 3.2592e-17 |  |
| Resilience_Total |  | - |  | Resilience_Control |  | 0.7083 | *** | 2.5213e-20 |  | 0.6942 | *** | 2.8041e-19 |  |
| Resilience_Total |  | - |  | Resilience_Challenge |  | 0.7151 | *** | 7.4764e-21 |  | 0.6751 | *** | 5.9286e-18 |  |
| Resilience_Total |  | - |  | Musical_Anhedonia |  | 0.1699 |  | 0.0582 |  | 0.1617 |  | 0.0717 |  |
| Resilience_Commitment |  | - |  | Resilience_Control |  | 0.1891 | * | 0.0347 |  | 0.2666 | ** | 0.0027 |  |
| Resilience_Commitment |  | - |  | Resilience_Challenge |  | 0.1443 |  | 0.1084 |  | 0.1928 | * | 0.0313 |  |
| Resilience_Commitment |  | - |  | Musical_Anhedonia |  | 0.0720 |  | 0.4248 |  | 0.0753 |  | 0.4041 |  |
| Resilience_Control |  | - |  | Resilience_Challenge |  | 0.2527 | ** | 0.0045 |  | 0.2280 | * | 0.0105 |  |
| Resilience_Control |  | - |  | Musical_Anhedonia |  | 0.0636 |  | 0.4813 |  | 0.0877 |  | 0.3311 |  |
| Resilience_Challenge |  | - |  | Musical_Anhedonia |  | 0.2022 | * | 0.0237 |  | 0.2161 | * | 0.0155 |  |
|  | | | | | | | | | | | | | |
| * p < .05, ** p < .01, *** p < .001 | | | | | | | | | | | | | |

**Table 4** Dispositional resilience (DRS-15) total and subscale mean scores

**_****__________________________________________________________________________________________________**

DRS-15 Non-Musicians (*N =* 61) Amateur Musicians (*N =* 64)

**___________________________________________________________________________________________________**

Commitment 11.70 (2.24) 11.88 (2.17)

Control 10.75 (2.39) 10.78 (2.44)

Challenge 9.16 (2.67) 10.31 (2.39)

DRS-15 total score 31.62 (4.84) 32.97 (4.92) **___________________________________________________________________________________________________**

Listed are the means (M) and standard deviations (SD) reported in brackets of the Norwegian revised DRS-15 questionnaire including three subscales: commitment, control, challenge, and DRS-15 total score. Each subscale has a highest possible score of 15 for a highest possible total score of 45.

**5.5 Resilience and BrainAGE scores**

Likewise, a direct comparison of resilience scores between the groups of non-musicians and amateur musicians (using an ANCOVA with BrainAGE scores as dependent variable, covariates: total resilience scores and years of education, fixed factors: musician status and gender) did not yield a significant group difference for the resilience scores (*F*(1,119) = 2.35; *p* = 0.13).

Spearman’s rank correlation~~s~~ was performed examining the relationship between BrainAGE and resilience scores (total scores). No correlations were found between BrainAGE score and the total resilience scores, *rs* = 0.06, *p* = 0.54, *N* = 125, including each subscale of the DRS-15 (See Table 5). Means and standard deviations between the groups for the different subscales of the DRS can be found in Table 6 below.

| Table 5. Correlation table for the association between BrainAGE and Resilience scores  Correlation Table | | | | | | | | | | | | | |
| --- | --- | --- | --- | --- | --- | --- | --- | --- | --- | --- | --- | --- | --- |
|  | | | | | | Pearson | | | | Spearman | | | |
|  | |  | |  | | r | | p | | rho | | p | |
| BrainAGE |  | - |  | Resilience_Total |  | 0.0741 |  | 0.4116 |  | 0.0557 |  | 0.5376 |  |
| BrainAGE |  | - |  | Resilience_Challenge |  | 0.1003 |  | 0.2658 |  | 0.1046 |  | 0.2459 |  |
| BrainAGE |  | - |  | Resilience_Control |  | -0.0154 |  | 0.8651 |  | -0.0213 |  | 0.8138 |  |
| BrainAGE |  | - |  | Resilience_Commitment |  | 0.0643 |  | 0.4760 |  | 0.0541 |  | 0.5493 |  |
| Resilience_Total |  | - |  | Resilience_Challenge |  | 0.7151 | *** | 7.4764e-21 |  | 0.6751 | *** | 5.9286e-18 |  |
| Resilience_Total |  | - |  | Resilience_Control |  | 0.7083 | *** | 2.5213e-20 |  | 0.6942 | *** | 2.8041e-19 |  |
| Resilience_Total |  | - |  | Resilience_Commitment |  | 0.6165 | *** | 1.9388e-14 |  | 0.6638 | *** | 3.2592e-17 |  |
| Resilience_Challenge |  | - |  | Resilience_Control |  | 0.2527 | ** | 0.0045 |  | 0.2280 | * | 0.0105 |  |
| Resilience_Challenge |  | - |  | Resilience_Commitment |  | 0.1443 |  | 0.1084 |  | 0.1928 | * | 0.0313 |  |
| Resilience_Control |  | - |  | Resilience_Commitment |  | 0.1891 | * | 0.0347 |  | 0.2666 | ** | 0.0027 |  |
|  | | | | | | | | | | | | | |
| * p < .05, ** p < .01, *** p < .001 | | | | | | | | | | | | | |

**Table 6** Dispositional resilience (DRS-15) total and subscale mean scores

**_****__________________________________________________________________________________________________**

DRS-15 Non-Musicians (*N =* 61) Amateur Musicians (*N =* 64)

**___________________________________________________________________________________________________**

Commitment 11.70 (2.24) 11.88 (2.17)

Control 10.75 (2.39) 10.78 (2.44)

Challenge 9.16 (2.67) 10.31 (2.39)

Total DRS score 31.62 (4.84) 32.97 (4.92) **___________________________________________________________________________________________________**

Listed are the means (M) and standard deviations (SD) reported in brackets of the Norwegian DRS-15 questionnaire including three subscales: commitment, control, challenge, and resilience total score.

**5.6 Lifestyle factors and BrainAGE scores**

A direct comparison of BrainAGE scores between the groups of active and non-active participants (using an ANCOVA with BrainAGE scores as dependent variable, covariates: years of education, fixed factors: exercise and gender) did yield a significant group difference for the BrainAGE scores (*F*(1,120) = 5.71; *p* = 0.02).

Similarly, a direct comparison of BrainAGE scores between the groups taking allergy medication and those who did not (using an ANCOVA with BrainAGE scores as dependent variable, covariates: years of education, fixed factors: allergy medication and gender) did yield a significant group difference for the BrainAGE scores (*F*(1,120) = 4.94; *p* = 0.03).

Likewise, a direct comparison of lifestyle factors (exercise) between the groups of non-musicians and amateur musicians (using an ANCOVA with BrainAGE scores as dependent variable, covariates: years of education, fixed factors: musician status, gender, and exercise) did yield a significant group difference for the BrainAGE scores (*F*(1,116*)* = 5.67, *p* = 0.02). See Fig. 8 for a violin plot visualizing the group differences in BrainAGE scores for those who are active and those who are not. The same analysis using allergy medicine as a fixed factor could not be run as sample size was too low.


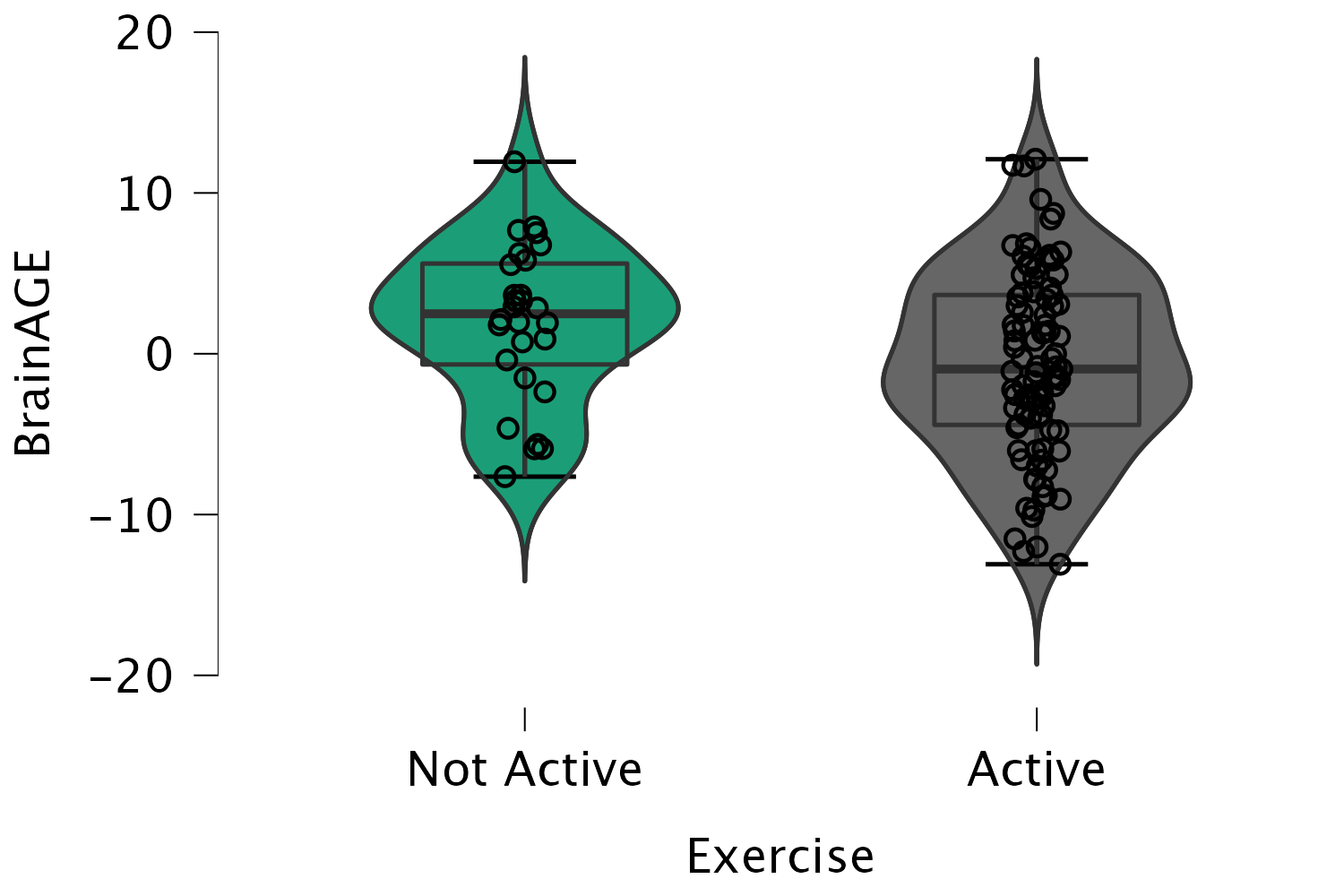


**Fig. 8** Distribution of the BrainAGE scores across all participants with regards to current engagement in physical activity. The distribution of BrainAGE scores (in years) is group-wise visualized as a violin plot showing the distribution of BrainAGE scores in healthy participants currently engaged in physical activity (active) versus individuals that are not (not active). The main horizontal line is the mean BrainAGE score of each group.
